# Machine learning for predicting acute lung injury after trauma: a systematic review and meta-analysis

**DOI:** 10.3389/fmed.2026.1882144

**Published:** 2026-07-29

**Authors:** Zhe Song, Yong Li, Bing Zhang, Sihan Pang, Weiyang Liu, Gongke Li

**Affiliations:** 1 Department of Intensive Care Medicine, The Affiliated Hospital of Yangzhou University, Yangzhou, Jiangsu, China; 2 Department of Emergency Medicine, The Affiliated Hospital of Yangzhou University, Yangzhou, Jiangsu, China; 3 Department of Trauma Medicine Center, The Affiliated Hospital of Yangzhou University, Yangzhou, Jiangsu, China

**Keywords:** acute lung injury, acute respiratory distress syndrome, machine learning, systematic review and meta-analysis, trauma

## Abstract

**Background:**

Acute lung injury (ALI) and acute respiratory distress syndrome (ARDS) remain major complications in trauma and other high-risk acute-care populations. Numerous statistical and machine-learning models have been developed for risk prediction, but their reported performance may vary because of differences in diagnostic criteria, predictor timing, validation strategies, and clinical settings. This systematic review and meta-analysis evaluated the performance of prediction models for ALI/ARDS and related acute pulmonary complications and explored potential sources of heterogeneity.

**Methods:**

The Cochrane Library, PubMed, EMBASE, and Web of Science were searched through August 13, 2025. Eligible studies developed or validated prediction models for ALI, ARDS, or related acute pulmonary complications in high-risk acute-care populations. Two reviewers independently screened studies, extracted data, and assessed risk of bias using PROBAST. Pooled C-index and accuracy were calculated when comparable estimates were available. Exploratory subgroup analyses examined diagnostic criteria, study design, and predictor timing. Sensitivity analyses included restriction to trauma- or burn-related studies and leave-one-out analyses.

**Results:**

Fourteen studies involving 5,608 participants were included. Outcome definitions varied and were based on the American-European Consensus Conference criteria, the Berlin definition, or other criteria. Logistic regression was the most commonly evaluated approach. In training datasets, pooled accuracy and C-index were 0.732 (95% CI, 0.694–0.768) and 0.80 (95% CI, 0.76–0.83), respectively. In test datasets, the corresponding estimates were 0.803 (95% CI, 0.756–0.843) and 0.81 (95% CI, 0.77–0.86). Penalized regression models, particularly LASSO, showed high discrimination in individual studies but were less frequently evaluated. Subgroup analyses suggested a lower pooled training C-index in studies using AECC criteria than in those using the Berlin definition or other criteria. Sensitivity analyses yielded results consistent with the primary findings.

**Conclusion:**

Prediction models for ALI/ARDS and related acute pulmonary complications showed generally good discrimination, with logistic regression remaining the most widely used approach. However, heterogeneous outcome definitions, inconsistent predictor timing, limited external validation, and incomplete reporting continue to hinder clinical translation. Future studies should standardize outcome definitions, clearly specify prediction time points and horizons, and prioritize transparent multicenter external validation.

**Trial registration:**

PROSPERO (Registration No. CRD42024576566).

## Introduction

Traumatic injury remains a major global health burden and is associated with substantial morbidity, mortality, and long-term disability. Patients with thoracic trauma, multiple injuries, hemorrhagic shock, massive transfusion, or prolonged mechanical ventilation are at increased risk of developing acute lung injury (ALI), acute respiratory distress syndrome (ARDS), or other acute pulmonary complications (1). In large trauma registries, ARDS occurs in approximately 1%−8% of patients, with substantially higher incidences reported in selected high-risk populations (2, 3). Although the incidence of post-traumatic ARDS has declined in some contemporary cohorts, mortality remains considerable (4). In certain high-risk settings, such as multiple-trauma patients at high altitude, the incidence may be even greater (5). These findings highlight the need for timely identification of patients at risk of clinically significant ALI after trauma.

Early risk prediction of ALI/ARDS is essential for patient stratification, timely preventive interventions, and optimal allocation of critical care resources (6, 7). Traditional prediction tools, including the Lung Injury Prediction Score (LIPS) and the Early Acute Lung Injury (EALI) score, have demonstrated clinical utility but are limited by modest positive predictive value and the requirement for numerous clinical variables (8). Therefore, more accurate and practical prediction models are needed. Importantly, the clinical usefulness of any prediction model depends not only on its discriminatory performance but also on whether its predictors are available before, or at the earliest stages of, lung injury development.

The terminology and diagnostic criteria for ALI and ARDS have evolved substantially over time. Earlier studies predominantly adopted the American-European Consensus Conference (AECC) definition, whereas more recent investigations have used the Berlin definition (9, 10). Although these definitions describe related clinical syndromes, they are not directly interchangeable, and previous studies have demonstrated differences in patient classification between the two criteria (11). In this review, we retained the outcome terminology reported in each original study, systematically extracted the diagnostic criteria used, and categorized studies according to the AECC definition, the Berlin definition, or other author-defined criteria. This approach enabled us to synthesize the available evidence while exploring the potential impact of diagnostic definitions on model performance. Machine learning methods have increasingly been applied to clinical prediction in trauma and critical care (12). Compared with conventional regression models, these approaches can incorporate complex and heterogeneous clinical information, including demographic characteristics, injury severity, physiological parameters, oxygenation and ventilatory variables, transfusion and resuscitation data, laboratory findings, imaging features, and biomarkers. However, many published models were developed using relatively small cohorts and lacked rigorous external validation, increasing the risk of overfitting and overly optimistic performance estimates. Furthermore, the complexity of some machine-learning models may reduce interpretability, limiting clinician confidence and hindering implementation in routine practice.

Interpretability is particularly important in ALI/ARDS prediction because clinicians must understand whether predictions are driven by biologically plausible and clinically actionable variables, such as injury severity, oxygenation status, transfusion exposure, inflammatory biomarkers, and resuscitation parameters (13). Transparent reporting of predictor selection, feature-selection strategies, and variable contributions is therefore essential for assessing the clinical applicability of prediction models.

Although an increasing number of studies have developed prediction models for ALI/ARDS and related acute pulmonary complications, the available evidence remains fragmented (14–17). Existing models differ considerably with respect to diagnostic definitions, study populations, predictor timing, feature-selection methods, data sources, and validation strategies (18). To date, no comprehensive systematic review has synthesized these methodological differences while quantitatively evaluating model performance across studies. Therefore, a systematic review and meta-analysis were conducted to evaluate the performance of prediction models for ALI/ARDS and related acute pulmonary complications in trauma and other high-risk acute-care populations. We also summarized diagnostic criteria, predictor acquisition timing, clinically relevant predictor categories, and feature-selection methods, and performed exploratory subgroup and sensitivity analyses to investigate important sources of clinical and methodological heterogeneity.

## Methods

### Eligibility criteria

Inclusion criteria were:

(1) Adults (≥18) from trauma- or burn-related cohorts, or from other high-risk acute-care or procedure-related populations, in whom ALI, ARDS, or related acute pulmonary complications were evaluated as the target outcome. Because relatively few prediction studies were conducted exclusively in trauma populations, eligible studies were subsequently classified as trauma/burn-related or non-trauma/procedure-related for prespecified sensitivity analyses.
(2) Studies published in English.
(3) Application of ML methods.
(4) Randomized controlled trials (RCTs), prospective cohort, nested case-control, and case-control studies.

Exclusion criteria were:

(1) Reviews, letters, case reports, meta-analyses, systematic reviews, meeting abstracts, and conference proceedings.
(2) Studies lacking performance metrics for predictive models, like sensitivity, specificity, accuracy, or confusion matrices.
(3) Studies unrelated to ALI or reporting only risk factors.
(4) Studies with incomplete or unavailable data.
(5) Animal studies.
(6) Studies focused on basic sciences, including pathology or physiology.
(7) ML studies with an insufficient sample size in the original dataset.
(8) Studies conducting only risk factor analysis without constructing an ML model.
(9) Studies that did not evaluate the constructed ML model.
(10) Studies with critical data errors.

### Literature search

Cochrane Library, PubMed, EMBASE, and Web of Science were thoroughly searched until August 13, 2025. Search terms combined Medical Subject Headings (MeSH) and entry terms, with no restrictions on geographic region.

EndNote X20 was employed to manage retrieved records, and duplicates were removed. Titles, abstracts, and full texts were screened for eligible studies. Literature search and screening were conducted independently by two reviewers, with discrepancies addressed by a third reviewer.

The strategy is detailed in Table S1.

### Data extraction

Data were systematically extracted as per the Modified PRISMA checklist. Extracted information encompassed the first author's name, publication year, duration of data collection, study design (prospective or retrospective), type of validation performed (external, internal, random split, or time split), and sample size, including total participants as well as the numbers in development and testing cohorts. Two independent reviewers performed data extraction, and any dissents were addressed by a third reviewer.

In addition, the reported outcome definition, diagnostic criteria, diagnostic-criteria group (AECC, Berlin definition, or other/author-defined criteria), predictor acquisition timing, predictor-timing group, predictor categories, representative predictors, and feature-selection methods were extracted for each included study. Predictor timing was classified as early when predictors were obtained at admission, within the first 24 h, or before outcome development, and as perioperative when predictors were collected before, during, or shortly after surgery or another clinical procedure. These characteristics are summarized in Table S2.

### Risk of bias assessment

The risk of bias was evaluated *via* the Prediction model Risk Of Bias Assessment Tool (PROBAST) (19), which is designed to assess bias throughout the meta-analytic workflow. Two reviewers independently conducted the assessment, and disagreements were adjudicated by a third reviewer.

### Statistical analysis

All analyses were enabled by R 4.5.0. Descriptive statistics were used to summarize study characteristics, model types, validation methods, predictor categories, and model performance. Performance metrics were synthesized separately for training and test datasets whenever available. Pooled C-index values were estimated using inverse-variance meta-analysis when effect estimates and corresponding measures of uncertainty were reported. Accuracy was pooled as a proportion. For studies reporting 95% confidence intervals, standard errors were derived from the reported confidence limits. When multiple prediction models were evaluated within the same study, model-specific estimates were extracted and synthesized according to model type. Exploratory subgroup analyses were performed according to diagnostic criteria (AECC, Berlin definition, or other/author-defined criteria), study design (prospective vs. retrospective), and predictor acquisition timing (early vs. perioperative). Given the limited number of studies in several subgroups, these analyses were considered exploratory and interpreted cautiously. Sensitivity analyses were carried out to assess the robustness of the pooled estimates. First, the primary analysis was repeated after restricting the dataset to trauma- or burn-related studies. Second, a leave-one-out analysis was performed by sequentially excluding individual studies and recalculating the pooled training C-index. Potential small-study effects were assessed visually using funnel plots. A funnel plot was generated for the primary meta-analysis of the training logistic regression (LR) C-index. In addition, an overall funnel plot including one primary estimate from each of the 14 studies was constructed and interpreted descriptively because different performance metrics and estimate types were combined. Egger's regression test was not performed when fewer than 10 homogeneous estimates were available or when heterogeneous performance metrics were pooled.

## Results

### Study selection

**Figure 1:**
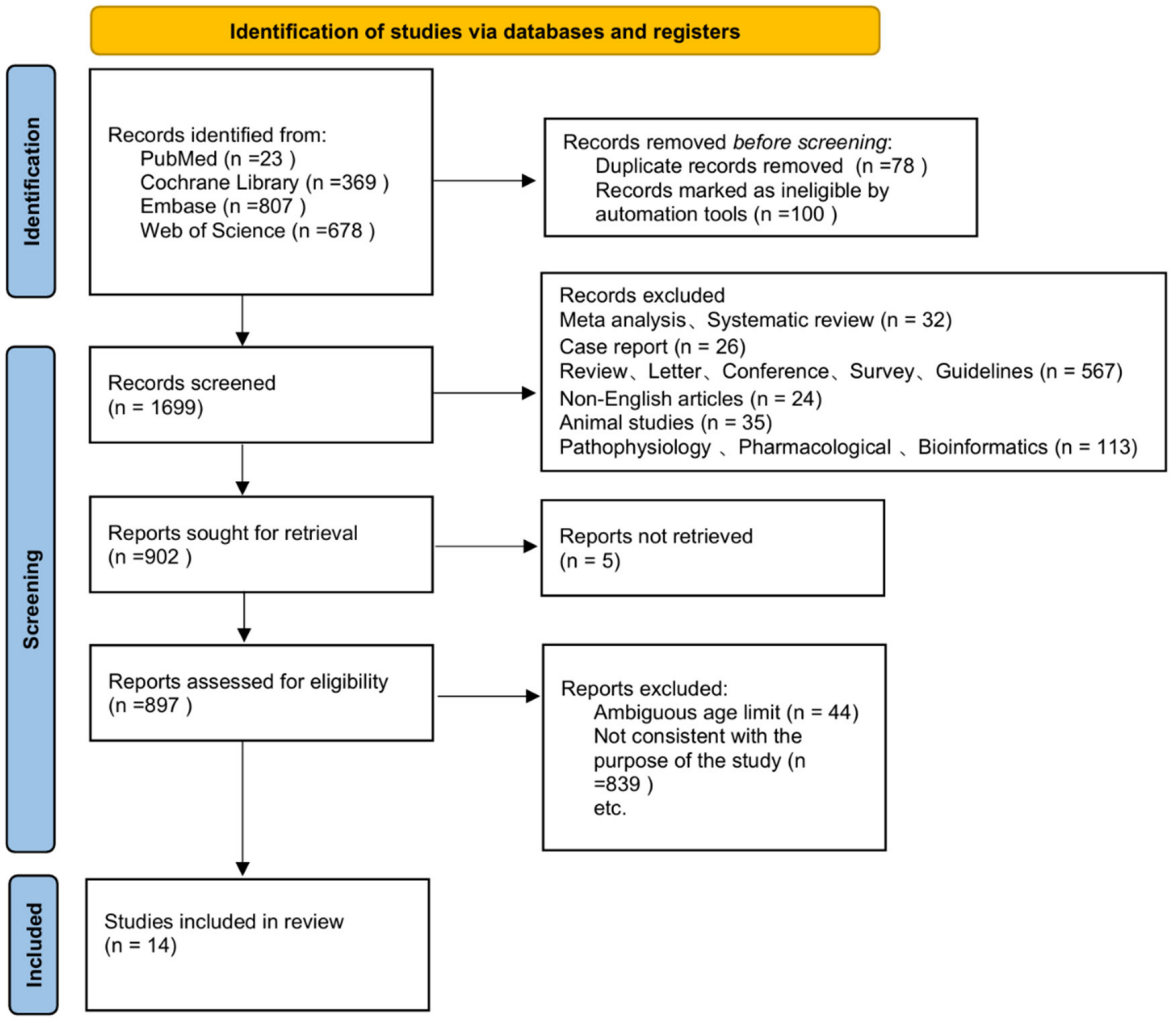
Flowchart of article screening.

1,877 articles were identified from multiple databases, including Cochrane (*n* = 369), PubMed (*n* = 23), Embase (*n* = 807), and Web of Science (*n* = 678). After 78 duplicates were removed, the titles and abstracts of 1,799 articles were screened, resulting in the inclusion of 14 studies. The PRISMA flow diagram of the selection process is provided in Figure 1.

### Characteristics of included studies

5,608 participants were encompassed, of whom 1,464 were allocated to the validation set. Data were collected from three countries. Among the encompassed studies, four were published within the last 5 years (2020–2025), indicating that research on machine learning (ML)-based prognostic models has emerged as a prominent area of interest and holds substantial clinical significance. In total, 17 prognostic models for ALI were identified, along with one externally validated model and five models validated through random sampling. The modeling approaches primarily comprised LR (*n* = 13), with Least Absolute Shrinkage and Selection Operator (LASSO), Best Subsets, Neural Network Model (NNM), and Multiple Models (MM), each applied once. Table 1 summarizes the characteristics of the encompassed studies.

**Table 1:** Basic information on the included literature.

References	Country	Type of study	Missing data	Events in the training dataset	Total number of samples in the training set	Verification set generation method	Events in the test dataset	Total number of samples in the test set	ML algorithm type	Number of model variables
Arabi et al. (20)	USA	Prospectively	—	51	109	—	—	—	LR	5
Afshar et al. (2)	USA	Prospectively	multiple imputation	38	113	multicenter study	27	104	LASSO, Best Subsets, LR	14
Cheng et al. (16)	China	Prospectively	—	81	127	—	—	—	LR	25
Howard et al. (21)	USA	Prospectively	—	91	272	random sampling	105	483	LR	12
Kinutani et al. (17)	Japan	Retrospectively	—	49	110	—	—	—	LR	26
Leng et al. (8)	China	Retrospectively	—	112	150	random sampling	112	1,022	LR	9
Liu et al. (24)	China	Prospectively	—	16	58	—	—	—	LR	12
Mark et al. (25)	USA	Prospectively	—	24	48	—	—	—	LR	32
Andrew et al. (26)	USA	Prospectively	multiple imputation	10	171	random sampling	14	171	LR	24
Pan et al. (14)	China	Prospectively	multiple imputation	11	34	random sampling	37	96	LR	42
Shah et al. (6)	USA	Prospectively	multiple imputation	93	283	—	—	—	LR	18
Tu et al. (7)	China	Retrospectively	multiple imputation	66	168	—	—	—	LR	33
Watkins et al. (22)	USA	Prospectively	multiple imputation	423	1762	random sampling	79	224	NNM, MM	29
Yuan et al. (15)	China	Prospectively	—	25	103	—	—	—	LR	16

LR, logistic regression; LASSO, least absolute shrinkage and selection operator; NNM, neural network model; MM, multiple models.

Regarding outcome definitions, seven studies used the AECC criteria, six used the Berlin definition, and one used other or author-defined diagnostic criteria. Eleven studies were prospective, and three were retrospective. Predictor acquisition was classified as early in nine studies and perioperative in five. Eight studies were conducted in trauma- or burn-related populations, whereas six involved non-trauma or procedure-related populations. Detailed information on outcome definitions, predictor timing, predictor categories, and feature-selection methods is provided in Table S2.

### Quality assessment

**Figure 2:**
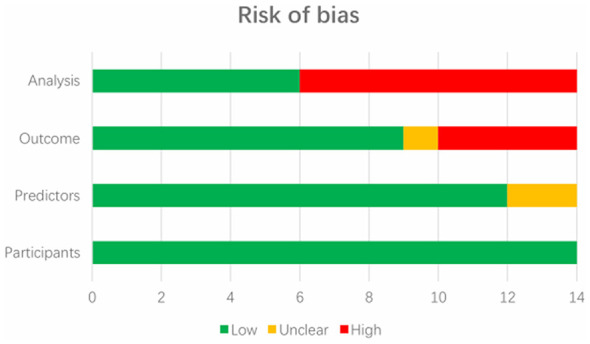
Risk of bias assessment.

The risk-of-bias evaluation revealed that 57.14% of studies were classified as high risk in the analysis domain, 28.57% as high risk in the outcome domain, 7.14% as medium risk in the outcome domain, and 14.29% as medium risk in the predictors domain (Figure 2).

### Training set and test set accuracy

**Figure 3:**
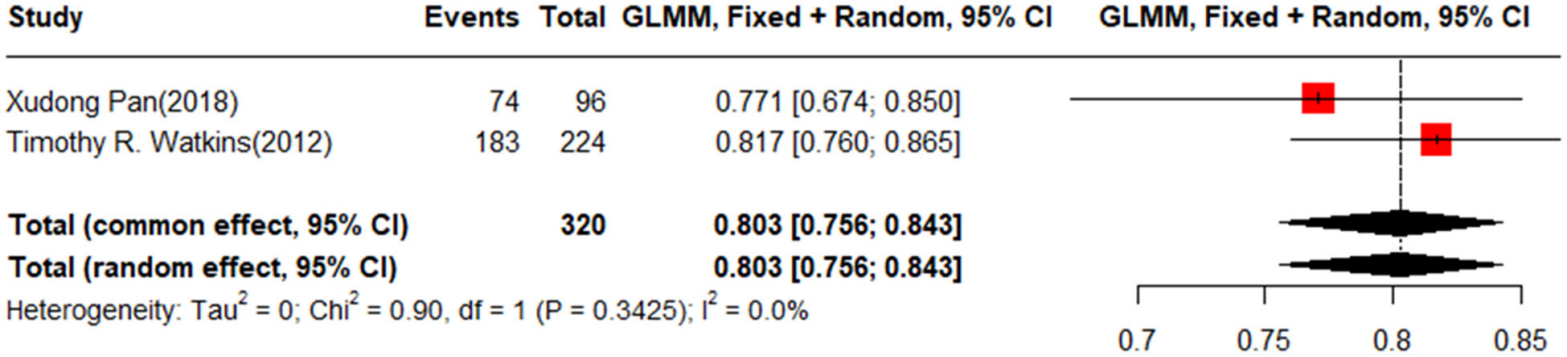
Test set accuracy.

In the test datasets, LR demonstrated strong predictive performance [*n* = 2, accuracy = 0.803 (95% CI: 0.756–0.843)] (Figure 3).

**Figure 4:**
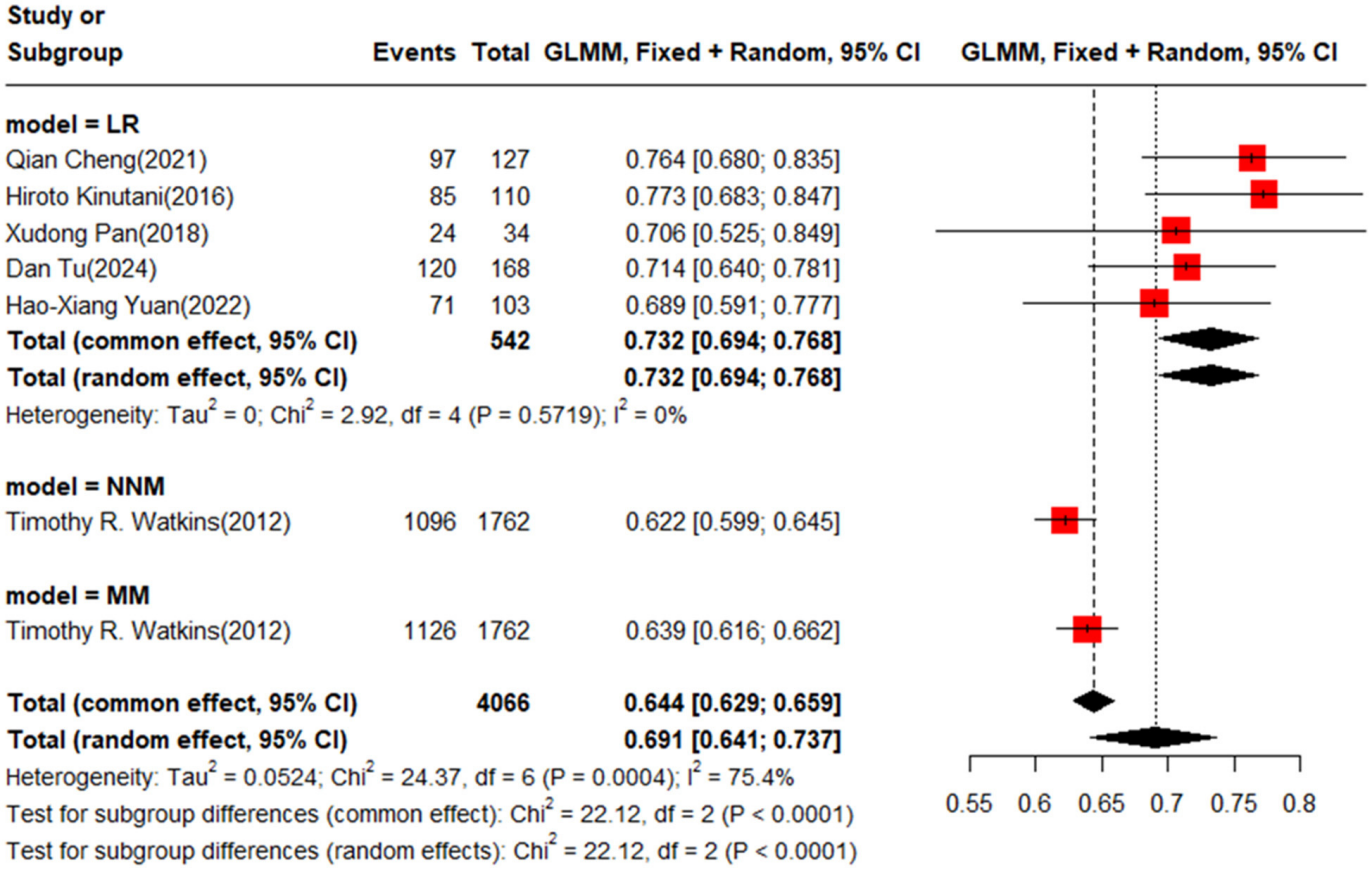
Training set accuracy.

In the training datasets, LR was both the most widely used and the highest-performing model [*n* = 5, accuracy = 0.732 (95% CI: 0.694–0.768)] (Figure 4). NNM and MM showed plain predictive value with accuracy of [*n* = 1, 0.622(0.599, 0.645)] and [*n* = 1, 0.639 (0.616, 0.662)], respectively (Table 2).

**Table 2:** Data analysis summary.

Performance metric	Model	Training					Test				
		*n*	Total	ACC	Low	Up	*n*	Total	ACC	Low	Up
Accuracy	LR	5	542	0.732	0.694	0.768	2	320	0.803	0.756	0.843
	MM	1	1,762	0.639	0.616	0.662	-	-	-	-	-
	NNM	1	1,762	0.622	0.599	0.645	-	-	-	-	-
	total	7	4,066	0.691	0.641	0.737	2	320	0.803	0.756	0.843
C-index	LR	12	1,575	0.800	0.760	0.830	5	1,938	0.810	0.770	0.860
	Best Subsets	1	113	0.910	0.860	0.970	1	113	0.890	0.830	0.950
	LASSO	1	113	0.920	0.870	0.980	1	113	0.900	0.840	0.960
	MM	1	1,762	0.650	0.630	0.680	-	-	-	-	-
	NNM	1	1,762	0.660	0.640	0.690	-	-	-	-	-
	total	16	5,325	0.770	0.720	0.840	7	2,164	0.850	0.800	0.900

LR, logistic regression; LASSO, least absolute shrinkage and selection operator; NNM, neural network model; MM, multiple models.

### Training set and test set C-index

**Figure 5:**
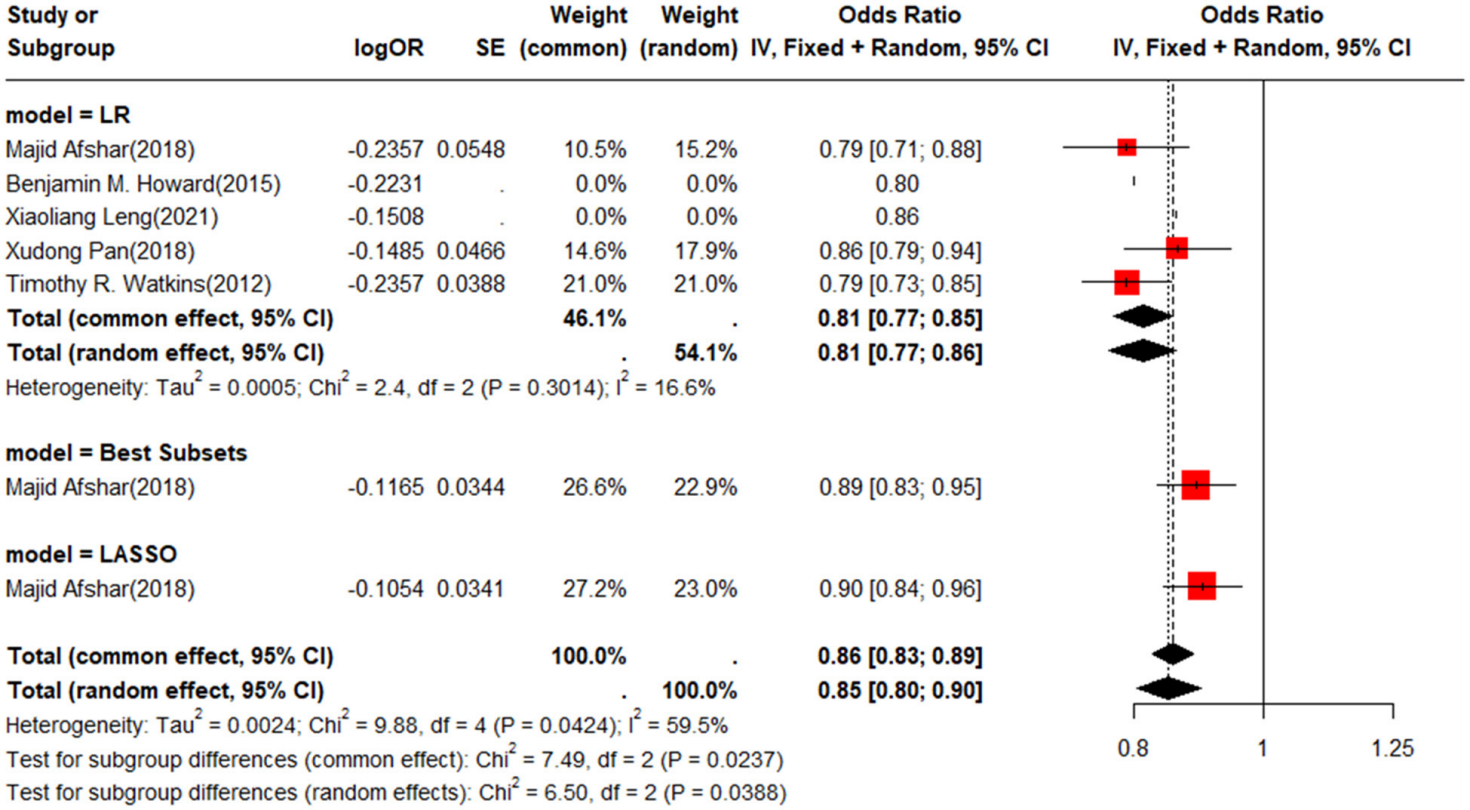
Test set C-index.

In the test datasets, LR remained the most frequently employed model [*n* = 5, C-index = 0.81 (95% CI: 0.77–0.86)], whereas LASSO achieved superior performance, with a C-index of 0.90 (95% CI: 0.84–0.96) despite a limited sample size [*n* = 1]. The Best Subsets model also showed strong predictive ability [*n* = 1, C-index = 0.89 (95% CI: 0.83–0.95)] (Figure 5).

**Figure 6:**
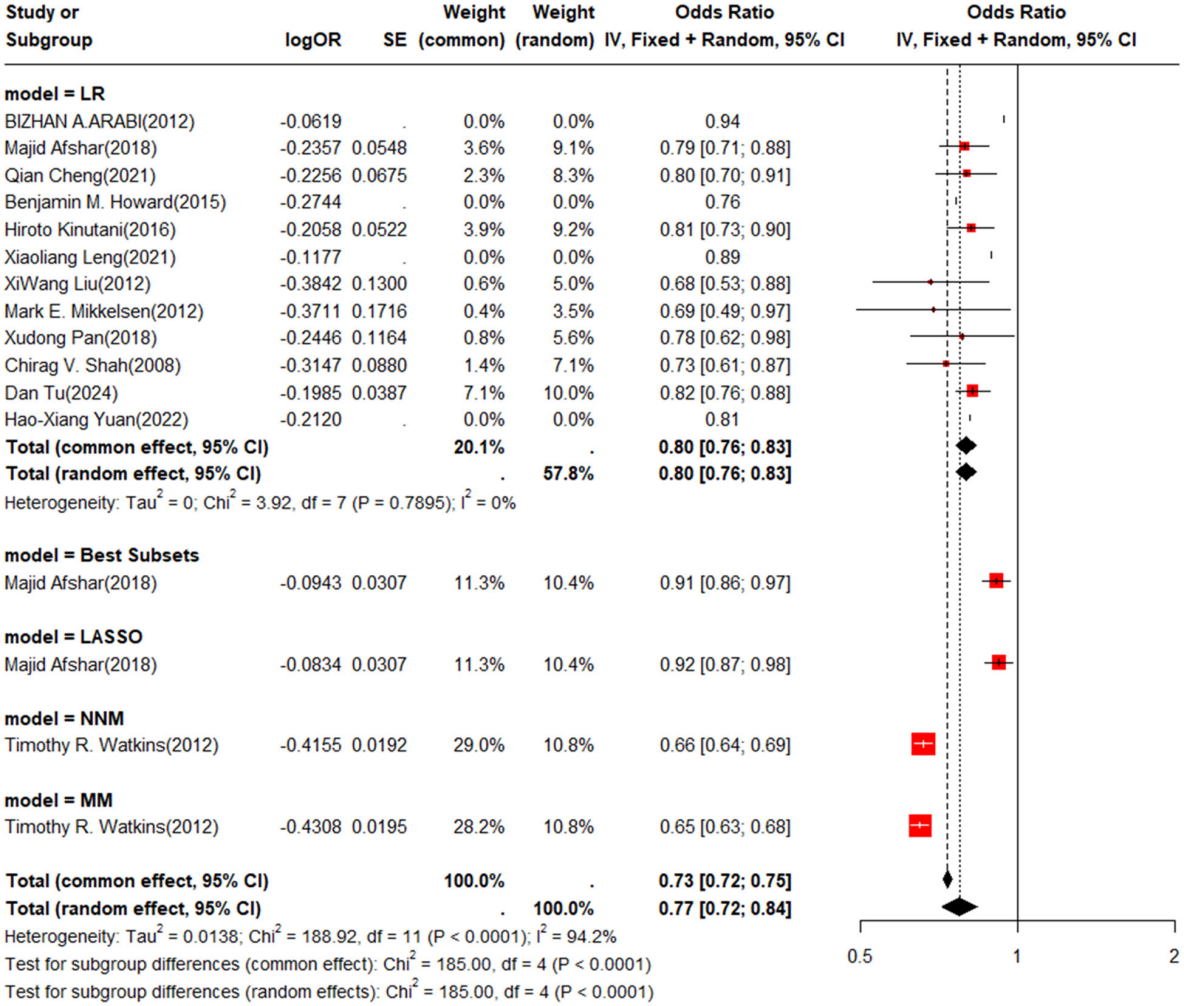
Training set C-index.

Within the training datasets, LR was again the most commonly applied model [*n* = 12, C-index = 0.80 (95% CI: 0.76–0.83)], whereas LASSO achieved the highest performance [*n* = 1, C-index = 0.92 (95% CI: 0.87–0.98)]. Best Subsets demonstrated consistent predictive value across models [C-index = 0.91 (95% CI: 0.86–0.97)]. NNM and MM showed plain predictive value with c-indices of [*n* = 1, 0.66 (0.64, 0.69)] and [*n* = 1, 0.65 (0.63, 0.68)] (Figure 6, Table 2).

### Subgroup and sensitivity analyses

Exploratory subgroup analyses were performed according to diagnostic criteria, study design, and predictor acquisition timing. For the training LR C-index, the pooled estimate was 0.807 (95% CI, 0.763–0.850) among studies using the Berlin definition, 0.709 (95% CI, 0.613–0.804) among those using the AECC criteria, and 0.810 (95% CI, 0.725–0.895) among studies using other or author-defined criteria (Figure S1A; Table S3). Stratification by study design yielded pooled training LR C-index values of 0.768 (95% CI, 0.716–0.820) for prospective studies and 0.817 (95% CI, 0.768–0.866) for retrospective studies (Figure S1B; Table S3). Training accuracy was broadly comparable across diagnostic-criteria and predictor-timing subgroups (Figures S1C, D; Table S3).

Sensitivity analyses demonstrated findings consistent with the primary analysis. Restricting the analysis to trauma- or burn-related studies produced a pooled training LR C-index of 0.797 (95% CI, 0.755–0.838), which was similar to the overall estimate (Figure S2; Table S3). Leave-one-out analysis showed that sequential exclusion of individual studies had minimal impact on the pooled training LR C-index, with estimates ranging from 0.779 to 0.799 (Table S4).

### Clinically relevant predictors

The most frequently reported predictor categories were demographic or baseline characteristics (13 studies), injury severity or injury pattern (8 studies), routine laboratory or coagulation variables (7 studies), respiratory, oxygenation, or ventilation variables (7 studies), and biomarkers related to inflammation or oxidative stress (7 studies). Transfusion or resuscitation variables, physiological or hemodynamic parameters, surgical or procedural factors, comorbidities, and imaging findings were reported less frequently (Figure S3, Table S2).

### Publication bias and small-study effects

Funnel plots were constructed to visually assess potential small-study effects. Because fewer than 10 homogeneous estimates were available for the primary meta-analysis of the training LR C-index, Egger's regression test was not performed to avoid an underpowered and potentially unreliable assessment (Figure S4A). An additional funnel plot including one primary estimate from each of the 14 studies was generated and interpreted descriptively because heterogeneous performance metrics and estimate types were combined (Figure S4B).

## Discussion

### Principal results

This systematic review and meta-analysis included 14 studies involving 5,608 patients from three countries and provides a comprehensive synthesis of prediction models for ALI/ARDS and related acute pulmonary complications. Logistic regression was the most frequently evaluated approach and demonstrated moderate-to-good discrimination and accuracy in both training and test datasets. Penalized regression methods, particularly LASSO regression, achieved high discriminatory performance in individual studies but were evaluated less frequently. Subgroup and sensitivity analyses indicated that the overall findings were robust and were not substantially influenced by any single study or by the inclusion of non-trauma/procedure-related populations. Nevertheless, studies using the AECC definition showed a numerically lower pooled C-index than those using the Berlin or author-defined criteria. Although this observation should be interpreted cautiously because of the limited number of studies in several subgroups, it highlights the potential impact of diagnostic definitions on reported model performance.

### Comparison with prior work

Previous research has demonstrated the potential of multidimensional ML algorithms for prognostic modeling in trauma populations. Nevertheless, the outcomes and patient cohorts differ from those examined in the present study. Rau et al. (18) evaluated multiple ML algorithms to estimate overall survival after trauma, Arabi et al. (20) employed neural networks and other models to predict mortality and length of stay in motor-vehicle collision victims, and Newgard et al. (3) modeled in-hospital survival within injured children treated at U.S. trauma centers. These studies collectively underscore that ML can outperform traditional scoring systems for general trauma prognosis, yet they primarily focus on mortality endpoints in heterogeneous or pediatric trauma populations.

This review differs in several key respects. First, it targets a specific organ-failure outcome, post-traumatic ALI or ARDS, rather than all-cause mortality. The incidence of ALI/ARDS is lower than that of mortality and is influenced by thoracic injury severity, transfusion practices, and ventilation strategies. Consequently, models trained to predict death possibly do not fully capture the nuanced physiologic and inflammatory pathways leading to lung failure, whereas the studies encompassed in this review often incorporated respiratory parameters and biomarkers. Second, the populations studied differ: Arabi et al.'s (20) dataset primarily comprised car-crash patients in Korea, Newgard et al. (3) analyzed pediatric trauma patients across 458 U.S. centers, and Rau et al. (18) utilized registry data spanning a wide range of injury mechanisms. In contrast, the present meta-analysis aggregated adult and mixed-age trauma cohorts with pulmonary outcomes, thereby increasing heterogeneity but enhancing generalizability (21).

Differences also emerged in modeling approaches and predictors. Prior trauma-mortality studies commonly employed random forests, gradient boosting, and neural networks, often concluding that neural networks yielded the highest discrimination, though LR remained competitive (22). In contrast, our synthesis revealed that traditional LR continues to be the most widely used algorithm for ALI/ARDS prediction, while penalized regression methods, like LASSO, can achieve superior performance. These distinctions possibly reflect the fact that lung injury depends on intricate inflammatory cascades and ventilatory parameters; incorporating high-dimensional predictors benefits from regularization and feature selection. Furthermore, many mortality-focused studies leveraged large administrative datasets with thousands of cases, enabling deep learning models to detect complex patterns, whereas several ALI/ARDS studies involved smaller cohorts, rendering simpler models more robust.

Variability in model performance likely reflects differences in outcome definitions, patient populations, predictor availability, and methodological approaches. Unlike mortality, which is a relatively objective endpoint, ALI/ARDS diagnosis depends on evolving consensus definitions and physiological criteria. Previous comparisons have demonstrated that patient classification differs between the AECC and Berlin definitions (9). Predictor availability immediately after injury also varied across studies. For example, some models used routinely available clinical and laboratory variables, whereas others incorporated biomarkers or procedure-related variables that may not be available at the intended prediction time point. These findings underscore the importance of transparent reporting and external validation, allowing clinicians to judge when and where a model may be applicable.

The timing of predictor acquisition is closely related to the intended clinical application of a prediction model. Models based on admission or early post-injury variables are better suited for early risk stratification and preventive intervention, whereas models incorporating perioperative or evolving physiological variables may be more appropriate for identifying developing or established lung injury. Although pooled accuracy was broadly comparable between early and perioperative models, these approaches address different clinical questions. Future studies should therefore clearly define the prediction time point, prediction horizon, and intended clinical use, whether for prevention, early warning, or diagnosis.

Several factors likely contribute to the observed differences in model performance. First, the type and breadth of predictors are crucial: models including physiological variables and inflammatory biomarkers better capture the pathobiology of lung injury compared with those relying solely on demographics or injury-severity scores. Second, sample size and event rate influence algorithm selection: small development cohorts limit the ability of complex algorithms (e.g., neural networks or gradient boosting) to learn stable patterns, often favoring simpler models. Third, feature-selection and regularization techniques (e.g., LASSO or ridge penalties) help mitigate overfitting by prioritizing the most informative variables. Fourth, hyperparameter tuning is essential for nonparametric methods like random forests, gradient boosting, or neural networks, yet tuning procedures are infrequently reported. Finally, handling missing data and class imbalance can affect model discrimination and should be standardized across studies.

Analysis of predictor categories showed that most models relied on clinically accessible variables, including demographic characteristics, injury severity, laboratory and coagulation indices, respiratory and oxygenation parameters, transfusion or resuscitation variables, and inflammatory biomarkers. These findings are biologically plausible because trauma-associated ALI/ARDS is driven by direct lung injury, systemic inflammation, endothelial dysfunction, hemorrhagic shock, transfusion exposure, and fluid imbalance. Models based primarily on routinely available clinical variables are likely to be more practical for bedside implementation, whereas biomarker-based models may provide incremental predictive value in selected clinical settings.

Despite encouraging predictive performance, substantial clinical and methodological heterogeneity remains. The included studies differed in patient populations, data sources, outcome definitions, predictor sets, feature-selection strategies, validation methods, and follow-up duration. Reporting of algorithm development, hyperparameter optimization, missing-data handling, and feature-selection procedures was often incomplete. Although statistical heterogeneity for the pooled logistic regression C-index was low, these methodological differences limit direct comparison across studies and reduce confidence in model generalizability. Future research should adopt standardized ALI/ARDS definitions, prespecify clinically relevant prediction windows, transparently report model development and feature-selection procedures, and prioritize multicenter external validation. Compared with other applications of machine learning in trauma research, ALI/ARDS prediction remains relatively underdeveloped. For example, a recent multicenter study of trauma-associated coagulopathy developed and externally validated machine-learning models using data from more than 13,000 patients across four centers (23). Similar methodological standards should be pursued for ALI/ARDS prediction.

Interpretability is another key issue for clinical translation. Many high-performing ML models are perceived as black boxes, which may reduce clinician confidence and limit adoption in emergency and critical care settings. Model-agnostic interpretation methods, such as SHAP and LIME, can improve transparency by quantifying both global feature importance and patient-specific predictor contributions. Incorporating these approaches into future studies may enhance clinician trust, facilitate model auditing, and support safe implementation in trauma and critical care practice.

## Limitations

This study has several limitations. First, only 14 studies met the inclusion criteria, and several subgroup analyses included relatively few estimates; consequently, subgroup findings should be regarded as exploratory. Second, considerable heterogeneity existed in diagnostic criteria, predictor timing, feature-selection methods, data sources, and validation strategies. Although these factors were examined through subgroup and sensitivity analyses where possible, residual heterogeneity remains unavoidable. Third, incomplete reporting of uncertainty measures limited quantitative synthesis for several performance metrics and may have reduced the precision of pooled estimates. Fourth, robust external validation, calibration assessment, decision-curve analysis, and detailed reporting of model implementation were lacking in many studies, limiting assessment of clinical applicability. Fifth, individual participant data were unavailable, precluding adjustment for important patient-level characteristics, including age, injury severity, comorbidities, transfusion exposure, and ventilation strategy. Finally, publication bias and small-study effects could not be formally assessed because homogeneous meta-analyses included fewer than 10 estimates and the overall funnel plot combined heterogeneous performance metrics.

## Conclusions

Prediction models for ALI/ARDS and related acute pulmonary complications demonstrated promising discriminatory performance, with logistic regression remaining the most extensively evaluated modeling approach. Penalized regression methods, particularly LASSO regression, also showed favorable discrimination but require validation in larger, multicenter cohorts. The current evidence is constrained by heterogeneous outcome definitions, inconsistent predictor timing, limited external validation, and incomplete reporting of feature-selection and interpretability methods. Future studies should adopt standardized ALI/ARDS definitions, use clinically meaningful predictors available at clearly defined prediction time points, and perform multicenter external validation before these models are implemented in routine trauma or critical care practice.

## Data Availability

The original contributions presented in the study are included in the article/Supplementary material, further inquiries can be directed to the corresponding author.

## Supplementary material

Table S1 Search strategy.

Table S2 Outcome definitions, diagnostic criteria, predictor acquisition timing, and feature-selection methods of the included studies.

Table S3 Subgroup and sensitivity analyses of pooled model performance.

Table S4 Leave-one-out sensitivity analysis of the pooled training C-index.

Figure S1 Subgroup analyses of model performance. **(A)** Forest plot of the training C-index stratified by diagnostic criteria. **(B)** Forest plot of the training C-index stratified by study design. **(C)** Forest plot of training accuracy stratified by diagnostic criteria. **(D)** Forest plot of training accuracy stratified by predictor acquisition timing.

Figure S2 Sensitivity analysis restricted to trauma- or burn-related studies.

Figure S3 Distribution of clinically relevant predictor categories across the included studies.

Figure S4 Funnel plots assessing potential small-study effects. **(A)** Funnel plot for the training logistic regression (LR) C-index. **(B)** Overall funnel plot including one primary estimate from each included study.
