# Supplementary material for: Machine learning for predicting acute lung injury after trauma: a systematic review and meta-analysis
Source: Front Med (Lausanne). 2026 Jul 29;13:1882144. doi: 10.3389/fmed.2026.1882144 (PMC13461909; doi:10.3389/fmed.2026.1882144)
Supplement: Supplementary file 1 [file Table_1.docx]

**Table S1 Search strategy**

| Database | Retrieval type | | Outcome | Time |
| --- | --- | --- | --- | --- |
| Cochrane | #1 | (Wounds and Injuries):ti,ab,kw OR (Injuries and Wounds):ti,ab,kw OR (Injuries, Wounds):ti,ab,kw OR (Wounds and Injury):ti,ab,kw OR (Injury and Wounds):ti,ab,kw | 8730 | 2025/8/13 |
|  | #2 | (Wounds, Injury):ti,ab,kw OR (Wounds):ti,ab,kw OR (Wounds):ti,ab,kw OR (Injuries):ti,ab,kw OR (Injury):ti,ab,kw | 86730 |  |
|  | #3 | (Research-Related Injuries):ti,ab,kw OR (Injury, Research-Related):ti,ab,kw OR (Research Related Injuries):ti,ab,kw OR (Research-Related Injury):ti,ab,kw OR (Physical Trauma):ti,ab,kw | 3472 |  |
|  | #4 | (Physical Traumas):ti,ab,kw OR (Trauma, Physical):ti,ab,kw OR (Trauma):ti,ab,kw OR (Traumas):ti,ab,kw | 21110 |  |
|  | #5 | #1 or #2 or #3 or #4 | 98732 |  |
|  | #6 | (machine learning):ti,ab,kw OR (Deep learning):ti,ab,kw OR (Prediction model):ti,ab,kw OR (Transfer Learning):ti,ab,kw OR (random forest):ti,ab,kw | 11819 |  |
|  | #7 | (artificial neural network):ti,ab,kw OR (ANN):ti,ab,kw OR (Support vector machine):ti,ab,kw OR (SVM):ti,ab,kw OR (Nomogram):ti,ab,kw | 4419 |  |
|  | #8 | (COX):ti,ab,kw OR (XGboost):ti,ab,kw OR (Logistic):ti,ab,kw OR (Decision tree):ti,ab,kw OR (c-index):ti,ab,kw | 56015 |  |
|  | #9 | (ROC):ti,ab,kw OR (AUC):ti,ab,kw OR (External validation):ti,ab,kw | 29464 |  |
|  | #10 | #6 or #7 or #8 or #9 | 92016 |  |
|  | #11 | (Acute Lung Injury):ti,ab,kw OR (Acute Lung Injuries):ti,ab,kw OR (Lung Injuries, Acute):ti,ab,kw OR (Lung Injury, Acute):ti,ab,kw OR (Injuries, Lung):ti,ab,kw | 3372 |  |
|  | #12 | (Injury, Lung):ti,ab,kw OR (Lung Injuries):ti,ab,kw OR (Pulmonary Injury):ti,ab,kw OR (Injuries, Pulmonary):ti,ab,kw OR (Injury, Pulmonary):ti,ab,kw | 7166 |  |
|  | #13 | (Acute Lung Injury):ti,ab,kw OR (Pulmonary Injuries):ti,ab,kw | 3414 |  |
|  | #14 | #11 or #12 or #13 | 7166 |  |
|  | #15 | #5 and #10 and #14 | 369 |  |
| Embase | #1 | ('wounds'/exp OR wounds) AND ('injuries'/exp OR injuries) OR (injuries:ti,ab,kw AND wounds:ti,ab,kw) OR 'injuries, wounds':ti,ab,kw OR (wounds:ti,ab,kw AND injury:ti,ab,kw) OR (injury:ti,ab,kw AND wounds:ti,ab,kw) OR 'wounds, injury':ti,ab,kw OR wounds:ti,ab,kw OR wound:ti,ab,kw OR injuries:ti,ab,kw OR injury:ti,ab,kw OR 'research-related injuries':ti,ab,kw OR 'injury, research-related':ti,ab,kw OR 'research related injuries':ti,ab,kw OR 'research-related injury':ti,ab,kw OR 'physical trauma':ti,ab,kw OR 'physical traumas':ti,ab,kw OR 'trauma, physical':ti,ab,kw OR trauma:ti,ab,kw OR traumas:ti,ab,kw | 1894588 | 2025/8/13 |
|  | #2 | machine learning software'/exp OR 'machine learning software' OR 'machine learning':ti,ab,kw OR 'deep learning':ti,ab,kw OR 'prediction model':ti,ab,kw OR 'transfer of learning':ti,ab,kw OR 'random forest':ti,ab,kw OR 'artificial neural network':ti,ab,kw OR 'health care management':ti,ab,kw OR 'support vector machine':ti,ab,kw OR nomogram:ti,ab,kw OR hip:ti,ab,kw OR xgboost:ti,ab,kw OR logistic:ti,ab,kw OR 'decision tree':ti,ab,kw OR 'c index':ti,ab,kw OR roc:ti,ab,kw OR auc:ti,ab,kw OR 'external validation':ti,ab,kw | 1462881 |  |
|  | #3 | acute lung injury'/exp OR 'acute lung injury' OR 'acute lung injuries':ti,ab,kw OR 'lung injuries, acute':ti,ab,kw OR 'lung injury, acute':ti,ab,kw OR 'injuries, lung':ti,ab,kw OR 'injury, lung':ti,ab,kw OR 'lung injuries':ti,ab,kw OR 'pulmonary injury':ti,ab,kw OR 'injuries, pulmonary':ti,ab,kw OR 'injury, pulmonary':ti,ab,kw OR 'pulmonary injuries':ti,ab,kw | 37265 |  |
|  | #4 | #1 AND #2 AND #3 | 807 |  |
| PubMed | #1 | ((((((((((((((((((Wounds and Injuries[MeSH Terms]) OR (Injuries[Title/Abstract] AND Wounds[Title/Abstract])) OR (Injuries, Wounds[Title/Abstract])) OR (Wounds[Title/Abstract] AND Injury[Title/Abstract])) OR (Injury[Title/Abstract] AND Wounds[Title/Abstract])) OR (Wounds, Injury[Title/Abstract])) OR (Wounds[Title/Abstract])) OR (Wound[Title/Abstract])) OR (Injuries[Title/Abstract])) OR (Injury[Title/Abstract])) OR (Research-Related Injuries[Title/Abstract])) OR (Injury, Research-Related[Title/Abstract])) OR (Research Related Injuries[Title/Abstract])) OR (Research-Related Injury[Title/Abstract])) OR (Physical Trauma[Title/Abstract])) OR (Physical Traumas[Title/Abstract])) OR (Trauma, Physical[Title/Abstract])) OR (Trauma[Title/Abstract])) OR (Traumas[Title/Abstract]) | 2,011,556 | 2025/8/13 |
|  | #2 | ((((((((((((((((((machine learning[MeSH Terms]) OR (machine learning[Title/Abstract])) OR (Deep learning[Title/Abstract])) OR (Prediction model[Title/Abstract])) OR (Transfer Learning[Title/Abstract])) OR (random forest[Title/Abstract])) OR (artificial neural network[Title/Abstract])) OR (ANN[Title/Abstract])) OR (Support vector machine[Title/Abstract])) OR (SVM[Title/Abstract])) OR (Nomogram[Title/Abstract])) OR (COX[Title/Abstract])) OR (XGboost[Title/Abstract])) OR (Logistic[Title/Abstract])) OR (Decision tree[Title/Abstract])) OR (c-index[Title/Abstract])) OR (ROC[Title/Abstract])) OR (AUC[Title/Abstract])) OR (External validation[Title/Abstract]) | 1,123,227 |  |
|  | #3 | ((((((((((Acute Lung Injury[MeSH Terms]) OR (Acute Lung Injuries[Title/Abstract])) OR (Lung Injuries, Acute[Title/Abstract])) OR (Lung Injury, Acute[Title/Abstract])) OR (Injuries, Lung[Title/Abstract])) OR (Injury, Lung[Title/Abstract])) OR (Lung Injuries[Title/Abstract])) OR (Pulmonary Injury[Title/Abstract])) OR (Injuries, Pulmonary[Title/Abstract])) OR (Injury, Pulmonary[Title/Abstract])) OR (Pulmonary Injuries[Title/Abstract]) | 13,596 |  |
|  | #4 | ((((((((((((((((((((Wounds and Injuries[MeSH Terms]) OR (Injuries[Title/Abstract] AND Wounds[Title/Abstract])) OR (Injuries, Wounds[Title/Abstract])) OR (Wounds[Title/Abstract] AND Injury[Title/Abstract])) OR (Injury[Title/Abstract] AND Wounds[Title/Abstract])) OR (Wounds, Injury[Title/Abstract])) OR (Wounds[Title/Abstract])) OR (Wound[Title/Abstract])) OR (Injuries[Title/Abstract])) OR (Injury[Title/Abstract])) OR (Research-Related Injuries[Title/Abstract])) OR (Injury, Research-Related[Title/Abstract])) OR (Research Related Injuries[Title/Abstract])) OR (Research-Related Injury[Title/Abstract])) OR (Physical Trauma[Title/Abstract])) OR (Physical Traumas[Title/Abstract])) OR (Trauma, Physical[Title/Abstract])) OR (Trauma[Title/Abstract])) OR (Traumas[Title/Abstract])) AND (((((((((((((((((((machine learning[MeSH Terms]) OR (machine learning[Title/Abstract])) OR (Deep learning[Title/Abstract])) OR (Prediction model[Title/Abstract])) OR (Transfer Learning[Title/Abstract])) OR (random forest[Title/Abstract])) OR (artificial neural network[Title/Abstract])) OR (ANN[Title/Abstract])) OR (Support vector machine[Title/Abstract])) OR (SVM[Title/Abstract])) OR (Nomogram[Title/Abstract])) OR (COX[Title/Abstract])) OR (XGboost[Title/Abstract])) OR (Logistic[Title/Abstract])) OR (Decision tree[Title/Abstract])) OR (c-index[Title/Abstract])) OR (ROC[Title/Abstract])) OR (AUC[Title/Abstract])) OR (External validation[Title/Abstract]))) AND (((((((((((Acute Lung Injury[MeSH Terms]) OR (Acute Lung Injuries[Title/Abstract])) OR (Lung Injuries, Acute[Title/Abstract])) OR (Lung Injury, Acute[Title/Abstract])) OR (Injuries, Lung[Title/Abstract])) OR (Injury, Lung[Title/Abstract])) OR (Lung Injuries[Title/Abstract])) OR (Pulmonary Injury[Title/Abstract])) OR (Injuries, Pulmonary[Title/Abstract])) OR (Injury, Pulmonary[Title/Abstract])) OR (Pulmonary Injuries[Title/Abstract])) | 454 |  |
|  | #5 | ((((((((((((((((((((Wounds and Injuries[MeSH Terms]) OR (Injuries[Title/Abstract] AND Wounds[Title/Abstract])) OR (Injuries, Wounds[Title/Abstract])) OR (Wounds[Title/Abstract] AND Injury[Title/Abstract])) OR (Injury[Title/Abstract] AND Wounds[Title/Abstract])) OR (Wounds, Injury[Title/Abstract])) OR (Wounds[Title/Abstract])) OR (Wound[Title/Abstract])) OR (Injuries[Title/Abstract])) OR (Injury[Title/Abstract])) OR (Research-Related Injuries[Title/Abstract])) OR (Injury, Research-Related[Title/Abstract])) OR (Research Related Injuries[Title/Abstract])) OR (Research-Related Injury[Title/Abstract])) OR (Physical Trauma[Title/Abstract])) OR (Physical Traumas[Title/Abstract])) OR (Trauma, Physical[Title/Abstract])) OR (Trauma[Title/Abstract])) OR (Traumas[Title/Abstract])) AND (((((((((((((((((((machine learning[MeSH Terms]) OR (machine learning[Title/Abstract])) OR (Deep learning[Title/Abstract])) OR (Prediction model[Title/Abstract])) OR (Transfer Learning[Title/Abstract])) OR (random forest[Title/Abstract])) OR (artificial neural network[Title/Abstract])) OR (ANN[Title/Abstract])) OR (Support vector machine[Title/Abstract])) OR (SVM[Title/Abstract])) OR (Nomogram[Title/Abstract])) OR (COX[Title/Abstract])) OR (XGboost[Title/Abstract])) OR (Logistic[Title/Abstract])) OR (Decision tree[Title/Abstract])) OR (c-index[Title/Abstract])) OR (ROC[Title/Abstract])) OR (AUC[Title/Abstract])) OR (External validation[Title/Abstract]))) AND (((((((((((Acute Lung Injury[MeSH Terms]) OR (Acute Lung Injuries[Title/Abstract])) OR (Lung Injuries, Acute[Title/Abstract])) OR (Lung Injury, Acute[Title/Abstract])) OR (Injuries, Lung[Title/Abstract])) OR (Injury, Lung[Title/Abstract])) OR (Lung Injuries[Title/Abstract])) OR (Pulmonary Injury[Title/Abstract])) OR (Injuries, Pulmonary[Title/Abstract])) OR (Injury, Pulmonary[Title/Abstract])) OR (Pulmonary Injuries[Title/Abstract])) | 23 |  |
| Web of Science | #1 | Wounds and Injuries (Topic) or Injuries and Wounds (Topic) or Injuries, Wounds (Topic) or Wounds and Injury (Topic) or Injury and Wounds (Topic) or Wounds, Injury (Topic) or Wounds (Topic) or Wound (Topic) or Injuries (Topic) or Injury (Topic) or Research-Related Injuries (Topic) or Injury, Research-Related (Topic) or Research Related Injuries (Topic) or Research-Related Injury (Topic) or Physical Trauma (Topic) or Physical Traumas (Topic) or Trauma, Physical (Topic) or Trauma (Topic) or Traumas (Topic) and Preprint Citation Index (Exclude – Database) | 5985261 | 2025/8/13 |
|  | #2 | machine learning (Topic) or machine learning (Topic) or Deep learning (Topic) or Prediction model (Topic) or Transfer Learning (Topic) or random forest (Topic) or artificial neural network (Topic) or ANN (Topic) or Support vector machine (Topic) or SVM (Topic) or Nomogram (Topic) or COX (Topic) or XGboost (Topic) or Logistic (Topic) or Decision tree (Topic) or c-index (Topic) or ROC (Topic) or AUC (Topic) or External validation (Topic) and Preprint Citation Index (Exclude – Database) | 3592662 |  |
|  | #3 | Acute Lung Injury (Topic) or Acute Lung Injuries (Topic) or Lung Injuries, Acute (Topic) or Lung Injury, Acute (Topic) or Injuries, Lung (Topic) or Injury, Lung (Topic) or Lung Injuries (Topic) or Pulmonary Injury (Topic) or Injuries, Pulmonary (Topic) or Injury, Pulmonary (Topic) or Pulmonary Injuries (Topic) and Preprint Citation Index (Exclude – Database) | 289599 |  |
|  | #4 | #1 AND #2 AND #3 and Preprint Citation Index (Exclude – Database) and Clinical Trial (Document Types) | 678 |  |
