# Supplementary material for: Machine learning for predicting acute lung injury after trauma: a systematic review and meta-analysis
Source: Front Med (Lausanne). 2026 Jul 29;13:1882144. doi: 10.3389/fmed.2026.1882144 (PMC13461909; doi:10.3389/fmed.2026.1882144)
Supplement: Supplementary file 2 [file Table_2.docx]

**Table S2. Outcome definitions, diagnostic criteria, predictor timing, and feature-selection methods of included studies**

| **Study** | **Reported outcome** | **Diagnostic criteria** | **Population / clinical setting** | **Predictor acquisition timing** | **Timing group** | **Predictor categories** | **Representative predictors** | **Feature-selection method** | **Comments** |
| --- | --- | --- | --- | --- | --- | --- | --- | --- | --- |
| BIZHAN A.ARABI (2012) | Pulmonary complications including ALI | AECC | Blunt traumatic spinal cord injury; North American Clinical Trials Network registry | Admission and early hospitalization data; admission neurological status and MRI features | Early | Demographics; injury mechanism; neurological severity; imaging characteristics | ASIA Impairment Scale grade, sex, age, injury mechanism, neurological level, lesion length/MRI findings | Univariable analysis followed by Bayesian logistic/negative binomial regression | ALI was one component of the broader pulmonary-complication outcome. |
| Majid Afshar (2018) | ARDS | Berlin definition | Burn injury or inhalation injury; prospective multicenter burn cohort | Clinical variables and plasma biomarkers collected within 24 h of admission | Early | Demographics; burn severity; inhalation injury; ventilator-related variables; biomarkers | %TBSA, inhalation injury, vWF-A2, age, sex, race, burn mechanism, alcohol/tobacco use, ICAM-1, sTNFr1, SP-D, IL-8 | Univariable comparisons; best subsets, stepwise logistic regression, and LASSO; bootstrap internal validation | Final parsimonious model included %TBSA, inhalation injury, and vWF-A2. |
| Qian Cheng (2021) | Trauma-associated ALI / ARDS | Berlin definition | Adult trauma patients recruited from the emergency department | Serum and clinical variables measured on ED admission within 24 h after trauma | Early | Demographics; injury severity; routine laboratory tests; blood gas; biomarker | CD5L, age, sex, WBC, hemoglobin, platelet, HCT, INR, bilirubin, creatinine, ALT, AST, APACHE II, GCS, ISS, blood gas variables | ROC analysis and logistic regression; univariable and multivariable models | Useful for reviewer comment about early prediction because predictors were obtained within 24 h of trauma. |
| Benjamin M. Howard (2015) | Hypoxemia and radiographically confirmed ARDS after trauma | Berlin definition | Intubated, critically injured trauma patients at an urban Level 1 trauma center | Prospectively collected early resuscitation and clinical data; patients with hypoxemia/ARDS in the first 24 h were excluded from main predictor models | Early | Demographics; injury severity; chest/head injury; transfusion; resuscitation volume | BMI, injury mechanism, head injury, chest injury, pRBCs, FFP, platelet transfusion, crystalloid, colloid, age, sex, ISS, GCS, ICU days | Univariable and multivariable logistic regression | Directly addresses early-prediction concern because the analysis aimed to ensure predictors preceded outcomes. |
| Hiroto Kinutani (2016) | Reperfusion pulmonary injury after balloon pulmonary angioplasty | procedure-related definition | Chronic thromboembolic pulmonary hypertension undergoing balloon pulmonary angioplasty | Periprocedural pulmonary arterial pressure measurements before and after BPA; CT evaluation 4 h after each session | Perioperative | Hemodynamics; pulmonary arterial pressure; angioplasty procedural variables | Pre/post-BPA distal pressure (Pd), proximal pressure (Pp), pressure ratio, PAP, CO, PVR, BNP, vessel/balloon variables | Univariable and multivariable logistic regression; ROC analysis | Not a conventional ALI/ARDS endpoint; retain as procedure-related pulmonary injury and discuss clinical heterogeneity. |
| Xiaoliang Leng (2021) | ALI after esophagectomy | Berlin definition | Esophageal cancer patients undergoing esophagectomy | Preoperative, intraoperative, anesthetic, laboratory, and postoperative ward data | Perioperative | Demographics; surgical/anesthetic variables; pulmonary function; routine laboratory tests; electrolytes | BMI, ASA class, DLCO%, duration of surgery, neutrophils, HDL-C, serum sodium, calcium, chloride | Univariable and multivariable logistic regression | Important example showing why ALI/ARDS definitions differ across surgical-ward settings. |
| XiWang Liu (2012) | Postoperative ALI after pediatric cardiac surgery | AECC | Infants and young children undergoing cardiac surgery with cardiopulmonary bypass | Perioperative serial sampling: before operation, before CPB, immediately after CPB, and 1, 12, and 24 h after operation | Perioperative | Demographics; cardiac surgical risk; oxygenation; mechanical ventilation; biomarkers | sRAGE, S100A12, sex, age, weight, SpO2, RACHS-1, ultrafiltrate volume, CPB time, operation time, MV duration, PaO2/FiO2 | Univariable and multivariable logistic regression | Pediatric cardiac-surgery population; contributes to clinical heterogeneity. |
| Mark E. Mikkelsen (2012) | ALI after major trauma | AECC | Major trauma patients admitted through the emergency department; nested case-control from a trauma cohort | Admission serum endocan measured before ICU admission; baseline clinical data from initial survey or first 24 h | Early | Demographics; injury severity; vital signs; routine labs; comorbidities; injury mechanism; transfusion/resuscitation; biomarker | Endocan, age, sex, ISS, APACHE III, temperature, heart rate, respiratory rate, MAP, WBC, hematocrit, platelet, creatinine, glucose, comorbidities, blunt/penetrating injury, transfusion, fluids | Univariable comparisons and multivariable logistic regression | Good candidate for early-prediction discussion because biomarker was measured on admission. |
| Andrew M. Naidech (2009) | ALI/ARDS after subarachnoid hemorrhage | AECC | Spontaneous aneurysmal or cryptogenic subarachnoid hemorrhage; non-trauma neurological cohort | Clinical variables collected prospectively; troponin measured on admission and the next day, and followed if abnormal | Early | Demographics; neurological severity; cardiac biomarkers; radiographic severity; transfusion; pulmonary complications | Cardiac troponin I, WFNS grade, Columbia CT score, ejection fraction, sex, smoking pack-years, coronary artery disease, PRBC transfusion, diagnostic PaO2/FiO2, pneumonia, vasospasm | Categorization of troponin quartiles and logistic regression | Non-trauma population; should be described as high-risk acute pulmonary injury setting if retained. |
| Xudong Pan (2018) | Preoperative ALI before Stanford type-A acute aortic dissection surgery | Berlin definition | Adults scheduled for Stanford type-A acute aortic dissection surgery | Preoperative and post-induction measurements; serum samples after induction; hemodynamic, echocardiographic, and inflammatory/oxidative biomarkers | Perioperative | Demographics; comorbidities; hemodynamics; echocardiography; routine labs; coagulation; inflammatory and oxidative stress biomarkers | Age, sex, BMI, smoking, hypertension, diabetes, symptom-to-surgery time, SBP/DBP/HR, aortic diameter, LVEF, LVEDD, EuroSCORE, Hb, WBC, glucose, TnI, FIB, FDP, D-dimer, APTT, PT, TF, TFPI, IL-6, IL-10, HLE, TNF-α, MDA, MPO, TAOC, TSOD, PLC, VEGF, PGI2, TXB2 | Univariable and multivariable logistic regression | Definition differs from Berlin/AECC full criteria; include as author-defined oxygenation-index based ALI. |
| Chirag V. Shah (2008) | ALI in critically ill trauma patients | AECC | Critically ill trauma patients admitted to ICU at a Level 1 trauma center | Baseline clinical variables collected from first 24 h; patients followed prospectively for ALI during first 5 days | Early | Demographics; injury severity; physiology; routine labs; transfusion; injury pattern; comorbidities | Age, sex, race, ISS, APACHE III, modified APACHE III, MAP, HR, hematocrit, bicarbonate, creatinine, blood units, blunt injury, long-bone fracture, pelvic fracture, pulmonary contusion, hypertension, diabetes | Multivariable explanatory logistic regression and causal pathway models | Strongly relevant to trauma-focused ALI analysis. |
| Dan Tu (2024) | ARDS in multiple trauma patients living in high-altitude areas | Berlin definition | Multiple trauma patients treated in a high-altitude hospital ICU | Demographics, treatment procedures, and injury severity collected on admission and during treatment | Early | Demographics; injury severity; trauma type; routine labs; transfusion/fluid therapy; ICU treatment variables; outcomes | ISS, thoracic injuries, APACHE II, trauma diagnosis, craniocerebral/limb/thoracic/abdominal trauma, time from injury to hospital, Hb, WBC, RBC, platelet, ALT, AST, bilirubin, blood loss, transfusion, fluid infusion, urine, mechanical ventilation days, ICU length of stay | Univariable logistic regression followed by multivariable logistic regression and risk-score construction | High-altitude criteria should be mentioned as an additional source of diagnostic heterogeneity. |
| Timothy R. Watkins (2012) | ARDS after major traumatic injury | AECC | Major trauma derivation cohort and independent multicenter validation cohort | Objective clinical variables available early after trauma; ED presentation and first 24 h variables | Early | Demographics; injury severity; physiology; injury pattern; transfusion/resuscitation; ventilation requirement | Age, APACHE II, ISS, GCS, ED hematocrit, lactate, INR, shock diagnosis, diabetes, alcohol use, blunt injury, femur fracture, pelvic fracture, flail chest, pulmonary contusion, crystalloid, massive/moderate RBC transfusion, FFP, cryoprecipitate, platelets, HPVC transfusion, mechanical ventilation | Bivariate screening, multivariable logistic regression with backward selection minimizing AIC, bootstrap internal validation, external validation | Key trauma prediction-model study; appropriate for subgroup and validation-strategy discussion. |
| Hao-Xiang Yuan (2022) | ALI after cardiac surgery with cardiopulmonary bypass | AECC | Adult cardiac surgery patients undergoing cardiopulmonary bypass | Microparticles and clinical variables measured before surgery, 12 h after surgery, and 3 d after surgery | Perioperative | Demographics; comorbidities; cardiac function; operative variables; microparticle size distribution | Microparticle size at peak, IQR, peak intensity, age, sex, BMI, drinking, smoking, hypertension, diabetes, hyperlipidemia, atrial fibrillation, coronary disease, prior MI, NYHA class, LVEF, CPB time, clamp time, operation time | Univariable and multivariable logistic regression; ROC analysis | Cardiac-surgery CPB population; reflects high-risk postoperative ALI rather than conventional trauma-only setting. |

Abbreviations: AECC, American-European Consensus Conference; ALI, acute lung injury; ARDS, acute respiratory distress syndrome; ICU, intensive care unit; ED, emergency department; MRI, magnetic resonance imaging; ASIA, American Spinal Injury Association; TBSA, total body surface area; vWF-A2, von Willebrand factor A2 domain; ICAM-1, intercellular adhesion molecule-1; sTNFr1, soluble tumor necrosis factor receptor 1; SP-D, surfactant protein D; WBC, white blood cell count; HCT, hematocrit; INR, international normalized ratio; ALT, alanine aminotransferase; AST, aspartate aminotransferase; APACHE II, Acute Physiology and Chronic Health Evaluation II; GCS, Glasgow Coma Scale; ISS, Injury Severity Score; BMI, body mass index; pRBCs/PRBC, packed red blood cells; FFP, fresh frozen plasma; BPA, balloon pulmonary angioplasty; CT, computed tomography; PAP, pulmonary arterial pressure; CO, cardiac output; PVR, pulmonary vascular resistance; BNP, brain natriuretic peptide; ASA, American Society of Anesthesiologists; DLCO, diffusing capacity of the lung for carbon monoxide; CPB, cardiopulmonary bypass; sRAGE, soluble receptor for advanced glycation end-products; RACHS-1, Risk Adjustment for Congenital Heart Surgery-1; MAP, mean arterial pressure; WFNS, World Federation of Neurological Surgeons; SBP, systolic blood pressure; DBP, diastolic blood pressure; HR, heart rate; LVEF, left ventricular ejection fraction; LVEDD, left ventricular end-diastolic diameter; Hb, hemoglobin; TnI, troponin I; FIB, fibrinogen; FDP, fibrin/fibrinogen degradation products; APTT, activated partial thromboplastin time; PT, prothrombin time; TF, tissue factor; TFPI, tissue factor pathway inhibitor; IL, interleukin; TNF-α, tumor necrosis factor-alpha; MDA, malondialdehyde; MPO, myeloperoxidase; TAOC, total antioxidant capacity; TSOD, total superoxide dismutase; VEGF, vascular endothelial growth factor; ROC, receiver operating characteristic; AIC, Akaike information criterion.
