# Supplementary material for: Machine learning for predicting acute lung injury after trauma: a systematic review and meta-analysis
Source: Front Med (Lausanne). 2026 Jul 29;13:1882144. doi: 10.3389/fmed.2026.1882144 (PMC13461909; doi:10.3389/fmed.2026.1882144)
Supplement: Supplementary file 3 [file Table_3.docx]

**Table S3. Subgroup and sensitivity analyses of pooled model performance**

| **Analysis** | **Subgroup** | **k** | **Pooled estimate (95% CI)** | **I^2^ (%)** | **P for heterogeneity** |
| --- | --- | --- | --- | --- | --- |
| Training LR C-index by diagnostic criteria | Berlin | 4 | 0.807 (0.763-0.850) | 0 | 0.932 |
| Training LR C-index by diagnostic criteria | Other/author-defined | 1 | 0.810 (0.725-0.895) | - | - |
| Training LR C-index by diagnostic criteria | AECC | 3 | 0.709 (0.613-0.804) | 0 | 0.891 |
| Training LR C-index by study design | Prospective | 6 | 0.768 (0.716-0.820) | 0 | 0.801 |
| Training LR C-index by study design | Retrospective | 2 | 0.817 (0.768-0.866) | 0 | 0.851 |
| Training LR accuracy by diagnostic criteria | Berlin | 3 | 0.733 (0.682-0.778) | 0 | 0.595 |
| Training LR accuracy by diagnostic criteria | Other/author-defined | 1 | 0.773 (0.685-0.842) | - | - |
| Training LR accuracy by diagnostic criteria | AECC | 1 | 0.689 (0.594-0.771) | - | - |
| Training LR accuracy by predictor timing | Early | 2 | 0.736 (0.682-0.783) | 0 | 0.34 |
| Training LR accuracy by predictor timing | Perioperative | 3 | 0.729 (0.67-0.781) | 0 | 0.375 |
| Sensitivity analysis: trauma/burn-related studies only | Overall | 5 | 0.797 (0.755-0.838) | 0 | 0.667 |
