# Supplementary material for: Machine learning for predicting acute lung injury after trauma: a systematic review and meta-analysis
Source: Front Med (Lausanne). 2026 Jul 29;13:1882144. doi: 10.3389/fmed.2026.1882144 (PMC13461909; doi:10.3389/fmed.2026.1882144)
Supplement: Supplementary file 4 [file Table_4.docx]

**Table S4. Leave-one-out sensitivity analysis for the pooled training C-index**

| **Omitted study** | **k remaining** | **Pooled C-index (95% CI)** | | **Change from primary estimate** |
| --- | --- | --- | --- | --- |
| Majid Afshar (2018) | 7 | 0.794 (0.755-0.834) | -0.003 | |
| Qian Cheng (2021) | 7 | 0.793 (0.755-0.831) | -0.004 | |
| Hiroto Kinutani (2016) | 7 | 0.790 (0.751-0.829) | -0.007 | |
| XiWang Liu (2012) | 7 | 0.799 (0.762-0.835) | 0.002 | |
| Mark E. Mikkelsen (2012) | 7 | 0.796 (0.760-0.832) | -0.001 | |
| Xudong Pan (2018) | 7 | 0.794 (0.758-0.831) | -0.003 | |
| Chirag V. Shah (2008) | 7 | 0.799 (0.762-0.836) | 0.002 | |
| Dan Tu (2024) | 7 | 0.779 (0.735-0.824) | -0.018 | |
